# An Unusual Presentation of Acute Appendicitis Mimicking Sigmoid Diverticulitis: A Case Report

**DOI:** 10.7759/cureus.74138

**Published:** 2024-11-21

**Authors:** Muhammed Salih Suer

**Affiliations:** 1 General Surgery, Ankara Etlik City Hospital, Ankara, TUR

**Keywords:** acute appendicitis, computerize tomography, diverticulitis, geriatric, surgery

## Abstract

Acute appendicitis typically causes right lower quadrant pain, but in elderly patients with comorbidities, it can present atypically, complicating diagnosis. This case highlights a rare presentation, mimicking sigmoid diverticulitis.

A 70-year-old man with chronic heart failure, arrhythmia, and renal failure presented with two days of left lower quadrant pain. Examination showed tenderness, rebound, and guarding. Laboratory tests revealed a white blood cell count of 12,000/µL and creatinine of 2.5 mg/dL. C-reactive protein was elevated at 25 mg/L. Computed tomography revealed an inflamed appendix with an appendicolith and localized inflammation near the sigmoid colon, but no diverticulitis. An appendectomy confirmed a perforated appendix in contact with the sigmoid colon.

In elderly patients, acute appendicitis may present atypically with left-sided pain, risking misdiagnosis. Careful evaluation and imaging are essential for accurate diagnosis and management in those with comorbidities.

## Introduction

Acute appendicitis commonly presents with right lower quadrant (RLQ) pain, though atypical presentations can complicate diagnosis. Misdiagnosis is particularly common in elderly patients due to nonspecific symptoms and the presence of multiple comorbidities [1]. Acute appendicitis remains a frequent cause of acute abdominal pain that often necessitates surgical intervention. However, its clinical presentation can vary widely, making diagnosis challenging [2]. This case study highlights an unusual presentation of acute appendicitis that mimics sigmoid diverticulitis and emphasizes the importance of clinical features, particularly physical examination findings, and diagnostic approaches for acute appendicitis.

Classically, acute appendicitis presents with initial periumbilical pain that later localizes to the RLQ of the abdomen, at McBurney's point. Physical examination often reveals rebound tenderness in the RLQ, indicating peritoneal irritation, where pain upon release of pressure is significant [3]. Additional physical signs include Rovsing’s Sign, where pain in the RLQ occurs upon palpation of the left lower quadrant (LLQ); Psoas Sign, which manifests as RLQ pain upon extension of the right hip, suggesting retrocecal appendicitis; and Obturator Sign, where pain occurs upon internal rotation of the right hip, indicating inflammation in contact with the obturator muscle. Systemic signs, such as fever and tachycardia, can also accompany appendicitis, though they are not specific [4].

Atypical presentations of appendicitis may occur due to anatomical variations in the position of the appendix. For example, retrocecal appendicitis may produce flank or back pain, rather than pronounced RLQ pain [5]. In elderly patients, symptoms may present more diffusely, such as with generalized abdominal pain and altered mental status, while pediatric patients may display irritability and refusal to eat [6].

Diagnostic approaches for acute appendicitis include laboratory tests and imaging. Leukocytosis with a left shift often supports the diagnosis, although it is not specific. C-reactive protein (CRP) levels may also be elevated, indicating inflammation, though they lack definitive diagnostic power [7]. Ultrasonography, particularly useful in pediatric and pregnant patients due to its safety, may show a non-compressible, dilated appendix with a diameter greater than 6 mm and the presence of an appendicolith. Computed tomography (CT) is considered the gold standard in adults, providing detailed images of an enlarged appendix and periappendiceal fat stranding, and identifying complications such as perforation [8]. When radiation exposure is a concern, such as in pregnancy, magnetic resonance imaging (MRI) can serve as an alternative, showing an enlarged appendix with surrounding inflammation [9,10]. Scoring systems, like the Alvarado Score and the Pediatric Appendicitis Score (PAS), incorporate clinical, physical, and laboratory findings to stratify the likelihood of appendicitis, aiding in diagnostic decisions and guiding the need for further imaging or surgical consultation [11,12].

## Case presentation

A 70-year-old male with a past medical history of chronic heart failure, arrhythmia, and renal failure presented to the Emergency Department with a two-day history of LLQ abdominal pain. He denied fever, nausea, vomiting, or changes in bowel habits. His past surgical history was unremarkable, and he had no history of constipation. The patient provided written informed consent for the use of his medical images and clinical information for academic and publication purposes, ensuring that privacy and anonymity are maintained.

Clinical findings

On examination, the patient was afebrile, with a heart rate of 88 bpm, blood pressure of 90/58 mmHg, and a respiratory rate of 18 breaths per minute. Abdominal examination revealed tenderness localized to the LLQ without rebound tenderness or guarding. Bowel sounds were present and normal. There were no signs of heart failure exacerbation.

Diagnostic assessment

**Figure 1:**
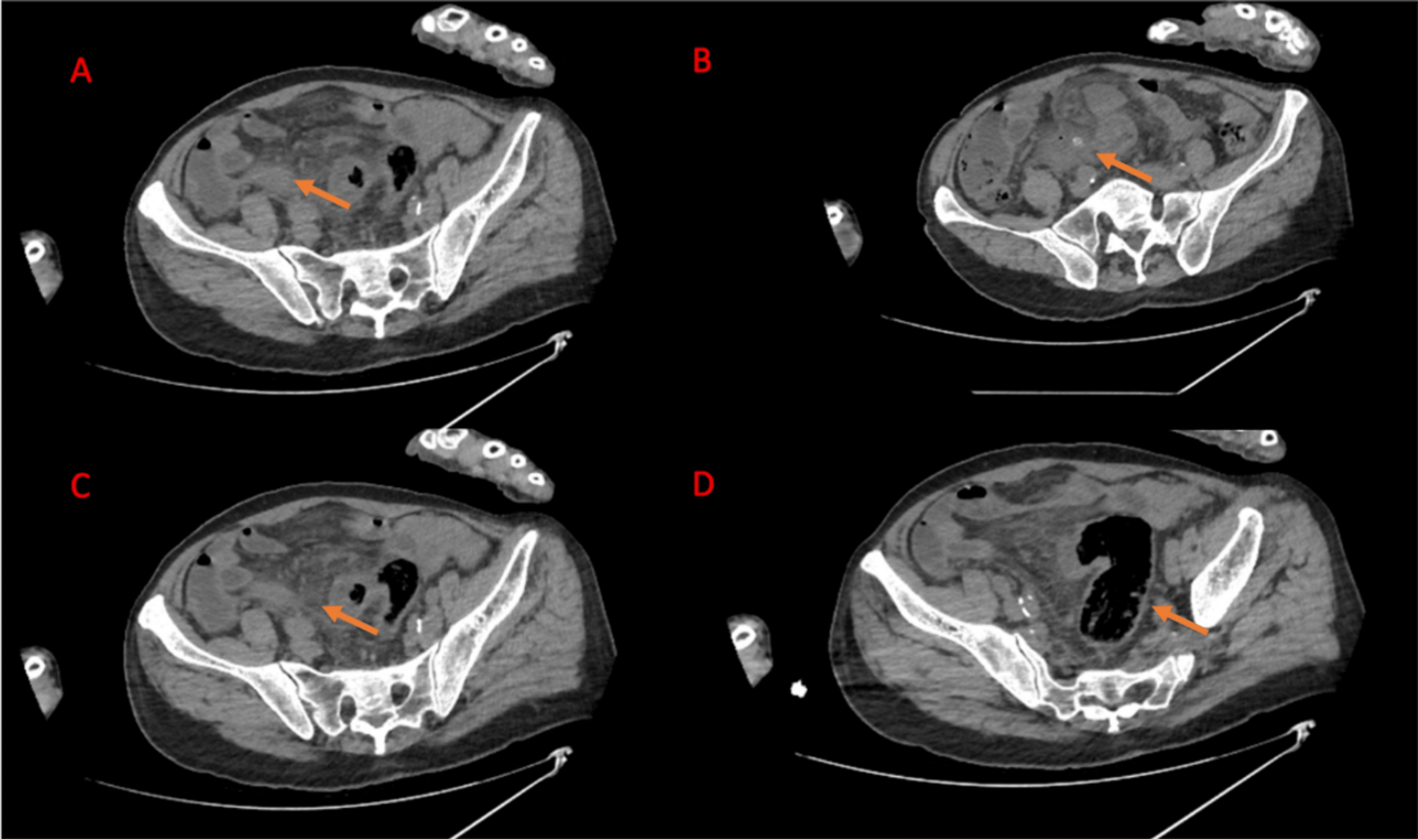
A) The appendix is inflamed and extended. B) An appendicolith is present in the pelvis, with surrounding tissue also exhibiting inflammation. C) and D) The sigmoid colon is inflamed, with no evidence of diverticular disease.

Laboratory tests revealed a white blood cell count of 12,000/µL, hemoglobin of 11 g/dL, and creatinine of 2.5 mg/dL. CRP was elevated at 25 mg/L. Urinalysis was unremarkable. An abdominal ultrasound was performed, which did not reveal any clear abnormalities. Due to the persistent pain and elevated inflammatory markers, a contrast-enhanced CT scan of the abdomen and pelvis was obtained. The CT scan revealed a thickened, inflamed appendix, suggestive of acute perforated appendicitis (Figure 1). Additionally, an appendicolith was observed outside of the appendix, free in the pelvis. There was no evidence of sigmoid diverticulitis, yet the sigmoid colon was inflamed, and the mesentery was dirty.

Surgery

**Figure 2:**
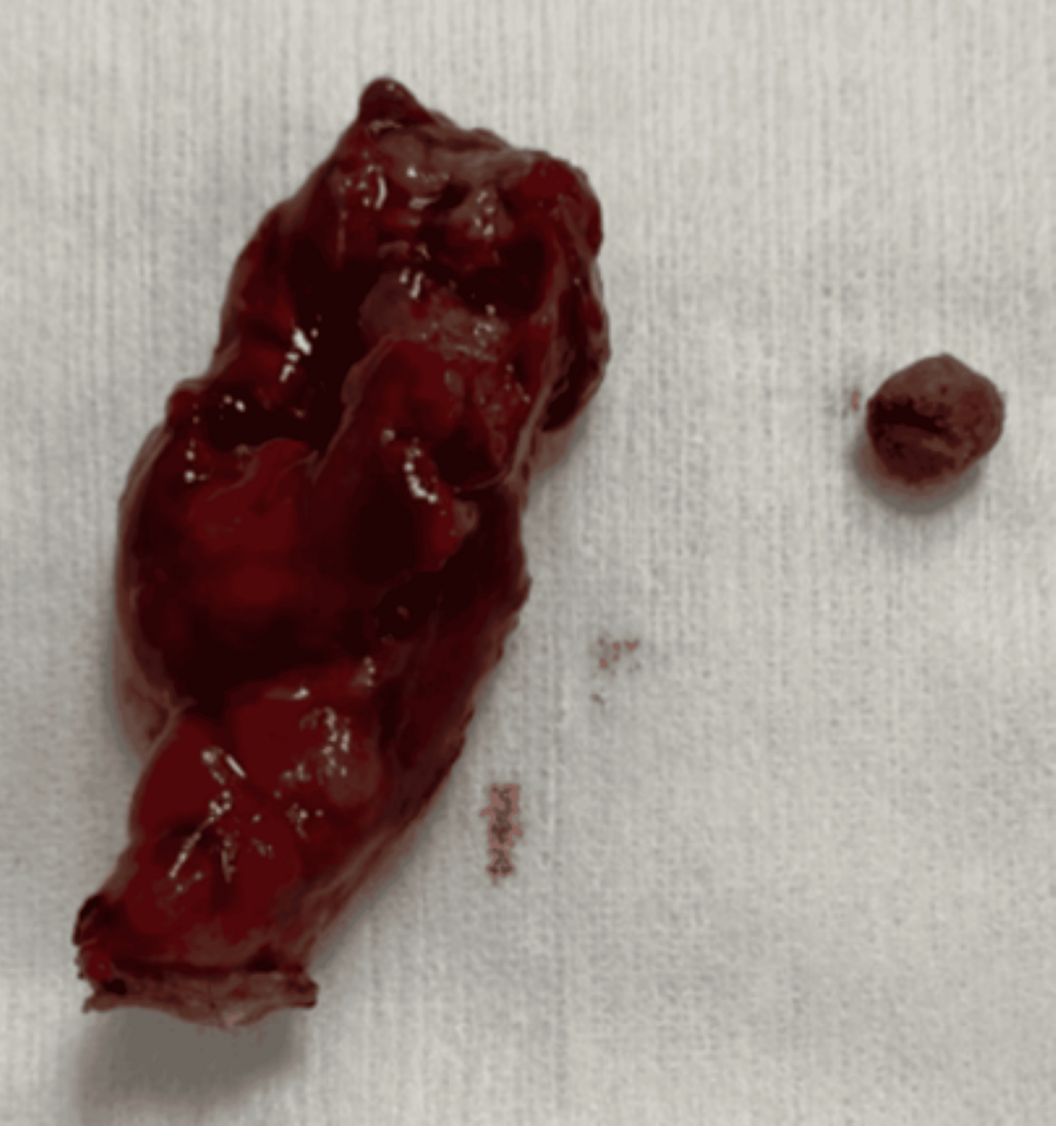
Acute perforated appendicitis with appendicolith.

The patient was admitted to the surgical unit for further management. Given his complex medical history and renal impairment, a multidisciplinary team, including cardiology and nephrology, was consulted. The patient underwent an appendectomy under general anesthesia, with careful intraoperative monitoring due to his cardiac status. An appendicolith was discovered in the pelvis (Figure 2). The appendix was found to be perforated, with the perforation in contact with the sigmoid colon, resulting in inflammation in the surrounding area. The inflammation resulted in a clinical presentation that was similar to diverticulitis.

Follow-up and outcomes

The postoperative course was unremarkable, and the patient was discharged on the third postoperative day. Pathological examination of the resected appendix confirmed acute appendicitis. At a follow-up visit two weeks later, the patient reported complete resolution of his abdominal pain and had no new symptoms or complications.

## Discussion

This case report presents an unusual presentation of acute appendicitis in an elderly patient with significant comorbidities, which clinically mimicked sigmoid diverticulitis. Atypical presentations of acute appendicitis are more prevalent in elderly patients, and comorbid conditions can obscure the classical symptoms, increasing the risk of misdiagnosis and delaying appropriate treatment [13,14]. In this case, the initial assessment was complicated by the presence of LLQ pain, a symptom commonly associated with diverticulitis. This example illustrates the diagnostic challenges that can arise when appendicitis presents atypically.

It is not uncommon for elderly patients with acute appendicitis to present with vague or non-specific symptoms. In this case, the patient presented with abdominal discomfort on the left side, which prompted an initial differential diagnosis that included sigmoid diverticulitis [15,16]. Such atypical locations of pain may result from variations in the anatomical position of the appendix or secondary inflammatory involvement of adjacent structures. It has been demonstrated that, in elderly patients, the classic RLQ pain may be absent or replaced by diffuse abdominal discomfort or pain in an atypical location, as observed in this patient's case. Furthermore, the presence of significant comorbidities, such as heart failure, arrhythmia, and renal failure in this patient, could have masked other systemic signs of infection, such as fever or marked leukocytosis, thereby further complicating the diagnosis.

## Conclusions

In elderly patients with complex medical histories, acute appendicitis can present with atypical symptoms, such as LLQ pain, which may be mistaken for other conditions, like sigmoid diverticulitis. To make an accurate diagnosis, it is essential to conduct a thorough clinical evaluation and perform appropriate imaging studies. This case study illustrates the importance of considering atypical presentations in differential diagnoses and the necessity of a multidisciplinary approach in managing patients with significant comorbidities.
